# Optimization of Flow-Cytometry Based Assay for Measuring Neutralizing Antibody Responses against Each of the Four Dengue Virus Serotypes

**DOI:** 10.3390/vaccines9111339

**Published:** 2021-11-17

**Authors:** Pragati Sharma, Kaustuv Nayak, Elluri Seetharami Reddy, Humaira Farooqi, Kaja Murali-Krishna, Anmol Chandele

**Affiliations:** 1 ICGEB-Emory Vaccine Center, International Centre for Genetic Engineering and Biotechnology, Aruna Asaf Ali Marg, New Delhi 110067, India; sharmapragati80@gmail.com (P.S.); kaustuvnayak@gmail.com (K.N.); rammku.rr@gmail.com (E.S.R.); 2 Department of Biotechnology, School of Chemical and Life Sciences, Jamia Hamdard, New Delhi 110062, India; 3 Kusuma School of Biological Sciences, Indian Institute of Technology Delhi, New Delhi 110016, India; 4 Department of Pediatrics, Division of Infectious Disease, Emory University School of Medicine, Atlanta, GA 30322, USA; 5 Emory Vaccine Center, Emory University School of Medicine, Atlanta, GA 30322, USA

**Keywords:** dengue, flowcytometry, neutralization, focus reduction neutralizing tests

## Abstract

Dengue is an important public health problem worldwide, with India contributing nearly a third of global dengue disease burden. The measurement of neutralizing antibody responses is critical for understanding dengue pathophysiology, vaccine development and evaluation. Historically, dengue virus neutralization titers were measured using plaque reduction neutralization tests (PRNTs), which were later adapted to focus reduction neutralization tests (FRNTs). Given the slow and laborious nature of both these assays, there has been interest in adapting a high-throughput flow cytometry based neutralization assay. However, flow cytometry based assays typically underestimate neutralization titers, and in situations where the titers are low they can even fail to detect neutralization activity. In this study, by evaluating graded numbers of input Vero cell numbers and viral inoculum, we optimized the flow cytometry based neutralization assay in such a way that it is sensitive and scores titers that are in concordance with focus reduction neutralization tests for each of the four dengue virus serotypes (*p* < 0.0001). Given that dengue is a global public health concern, and several research groups are making efforts to understand its pathophysiology and accelerate vaccine development and evaluation both in India and worldwide, our findings have timely significance for facilitating these efforts.

## 1. Introduction

Dengue is a mosquito-borne human viral disease caused by any of the four dengue virus serotypes (DENV1, DENV2, DENV3, DENV4) that are currently present in over 150 countries and continue to expand globally [1,2]. It is estimated that dengue viruses can infect over 390 million people each year resulting in over 100 million clinical cases, with clinical symptoms ranging from febrile fever to dengue hemorrhagic fever (DHF) and/or dengue shock syndrome (DSS), which can be fatal, especially among children [2,3]. India contributes to nearly a third of global dengue disease burden and thus vaccination or other intervention strategies are urgently needed [1,4]. Several vaccine candidates are at different stages of development and/or clinical trials in India and worldwide with the goal of eliciting a balanced immune response with broadly neutralizing antibodies against all four dengue serotypes [5,6]. These efforts require the measurement of neutralizing antibody responses against each of the four dengue virus serotypes in a large set of samples from dengue-exposed and/or vaccinated populations [7,8].

Traditionally, neutralizing antibodies against dengue viruses have been evaluated using plaque reduction neutralization tests (PRNTs). These tests rely on infecting monolayers of cell lines such as Vero cells at a very low multiplicity of infection (MOI), typically at an MOI of 0.01. The monolayers are then overlaid with agarose or methyl cellulose in such a way that the virus undergoes multiple rounds of replication and lyses the cells within the area of the first infected cell. The plaques formed are then counted in serially diluted plasma to estimate PRNT_50_, the dilution at which 50% of the virus is neutralized [9,10,11]. During the past decade, several researchers have adapted a modified method of the PRNT, called focus reduction neutralization tests (FRNTs). FRNTs rely on the detection of infected foci by enzyme-linked immunospot (ELISPOT) rather than the detection of plaques [12,13]. This method offers several advantages over the PRNT, especially in situations where the infecting virus fails to form visible plaques. Although generally considered to be gold-standard assays, both these methods are low throughput and laborious in nature since they typically require 3–6 days to allow for multiple rounds of viral replication in order for visible plaque or foci formation, thereby making it difficult to employ these in situations such as vaccine trials or population-based studies where a large number of samples need to be screened.

Hence, there has been great interest in developing high-throughput assays for measuring neutralizing antibody responses against dengue viruses [7,8]. Martin et al. reported an immunocytometric assay using Raji cells expressing DC-SIGN (CD206) [14]. Additionally, there have been efforts to assay dengue-neutralizing titers using reporter viruses [15]. However, infection with live dengue viruses offers an additional advantage over reporter viruses because they can be adapted to primary viral isolates and can measure antibody responses directed to other proteins that reporter viruses may not necessarily encode. Accordingly, Kraus et al. developed a 96-well plate flow cytometry based neutralization assay using live dengue viruses and U937 cells expressing DC-SIGN [7].

Although several researchers are beginning to use flow-based assays for measuring neutralizing antibody responses against dengue viruses [16,17], so far there is limited information pertaining to optimal assay conditions for flow cytometry based assays in such a way that the titers measured by the assay are in concordance with the titers calculated by the FRNT. Two published studies indicated that the titers obtained by flow-cytometry based assay tend to be lower than those obtained by PRNT or FRNT [7,17]. Bearing these points in mind, in this study we addressed the following questions: How well do the neutralizing antibody titers calculated by flow cytometry based assay correlate with titers obtained by the conventional FRNTs? Can we optimize the flow-based neutralizing assay such that the neutralization titers obtained by this assay are in concordance with the traditional foci-based neutralizing assay?

## 2. Material and Methods

### 2.1. Cell Lines

Vero cells (ATCC number: CCL-81) were used for both foci-based and flow cytometry based neutralization assays. Cells were maintained in Dulbecco’s modified Eagle’s medium (DMEM) supplemented with 10% heat-inactivated FBS, penicillin G (100 IU/mL), streptomycin (100 ug/mL), and 2 mM L-glutamine.

### 2.2. Viral Growth and Titration

Stocks of dengue virus 1 (West Pac/74-Nauru 1974, accession number U88536.1), dengue virus 2 (S-16803-Thailand 1974, accession number GU289914.1), dengue virus 3 (CH 53489-Thailand 1973, accession number DQ863638.1), and dengue virus 4 (TVP-360/S341750-Colombia 1982, accession number KU513442.1) were prepared by inoculating C6/36 (ATCC number: CRL-1660) monolayers in a 75 cm^2^ tissue culture flask with 0.01 MOI of virus diluted in 1 mL of DMEM–2% FBS as described previously [18]. After 2 h, 10 mL of DMEM supplemented with 2% FBS was added, and the cells were cultured for 7 days at 28 °C in 5% CO_2_. The supernatant was then removed from the cells and centrifuged for 10 min at 4000 rpm to pellet down cellular debris. The viruses were stored in aliquots at −80 °C and titrated using focus forming assay as described previously [9,10,18].

### 2.3. Monoclonal Antibodies

The flavivirus group-reactive monoclonal antibody 4G2 was purchased from Millipore (clone D1-4G2-4-15; #MAB10216) and is known to neutralize all four serotypes of the dengue virus [19,20]. A 2H2 clone of monoclonal antibody known to bind all four dengue serotypes [21,22] was purchased from Millipore (clone D2-2H2-9-21; #MAB8705). This antibody was conjugated with an Alexa Fluor 488 microscale protein kit (Invitrogen; #A30006).

### 2.4. Plasma Samples

Residual plasma (*n* = 20) was obtained from buffy coats of healthy blood bank donors that were negative for HIV 1-2, HBV, and HCV by nucleic acid amplification test (NAT) and positive for dengue binding by ELISA [13]. The plasma was aliquoted and stored at −80 °C. The study was approved by an institutional ethical clearance committee (ICGEB/IEC/2019/09, version iii).

### 2.5. Focus Reduction Neutralization Test

Focus reduction neutralization tests (FRNTs) were performed as described previously [12,13]. Briefly, Vero cells were seeded at a concentration of 15,000 cells/well in a 96-well flat-bottom plate overnight. Serially diluted (1:25 to 1:2,048,000) heat-inactivated plasma samples or dengue monoclonal antibody 4G2 were incubated with 150 plaque forming units (PFU) of dengue virus for 1 h at 37 °C with 5% CO_2_. The virus–plasma mixture was transferred onto Vero cells and they were incubated for 1 h at 37 °C with 5% CO_2_. Then, 2% (wt/vol) methylcellulose (Sigma; #M0512-2506) with ciprofloxacin and amphotericin B overlay was applied. After 3 days of incubation at 37 °C with 5% CO_2_ the cells were washed with PBS and fixed with an ice-cold 1:1 mixture of acetone and methanol. Foci were stained using 4G2 antibody for 2 h followed by HRP-linked anti-mouse IgG (Cell Signaling; 7076S) for 1 h and developed using TrueBlue Peroxidase substrate (KPL; #5078-02). FRNT_50_ was determined as plasma dilution required for 50% reduction of the viral PFU compared to the control well. All samples with FRNT_50_ at 1:50 or below were scored as non-neutralizing.

### 2.6. Flow Cytometry Based Neutralization Assay

The flow cytometry based neutralization test was performed as described earlier unless otherwise indicated [7,8]. Briefly, Vero cells were seeded at 50,000 cells per well in 96-well flat-bottom plates. Plasma samples were heat inactivated at 56 °C for 30 min. The plasma or 4G2 monoclonal antibody was serially diluted twofold starting at 1:25 for plasma or 20 µg/mL for 4G2 monoclonal antibody respectively in DMEM supplemented with 2% FBS, penicillin, and streptomycin. Fifty thousand PFU of dengue virus was then added to the diluted plasma or 4G2 antibody to achieve an MOI of 1 and incubated at 37 °C in 5% CO_2_ for 1 h. In situations where the input Vero cell number per well was altered, the PFU of the dengue virus was also altered such that the MOI always remained constant at 1. The virus–antibody mixture was then added to the Vero cells. The plates were incubated at 37 °C in 5% CO_2_ for 2 h after which an additional 100 µL of DMEM containing 2% FBS was added to each well, and the plates were further incubated at 37 °C in 5% CO_2_ for 24 h. For staining, first, culture media were aspirated and saved in a duplicate U-bottom plate. The cells were then washed with 50 µL of PBS followed by incubation with 50 µL of trypsin-EDTA for 2 min. The trypsin was neutralized by adding the saved culture media and then transferred back to the duplicate U-bottom plate. The cells were then thoroughly washed with media followed by fixation (eBiosciences, San Diego, CA, USA; #00-8222) and permeabilization and stained with FITC-conjugated 2H2 clone of monoclonal antibody. Cells were acquired in BD LSR Fortessa X-20 flow cytometry (BD Biosciences, San Jose, CA, USA) using rapid high-throughput screening (HTS). The percentage of cells positive for staining with FITC-conjugated 2H2 clone in the virus-alone well was considered as 100%, and the plasma dilution that resulted in 50% reduction from virus alone was considered as the 50% neutralization titer (FRNT_50_).

### 2.7. Statistical Analyses

Nonlinear dose–response regression analysis was performed to calculate 50% neutralization. Correlation analysis was performed using Pearson correlation coefficient. Both analyses were performed using Prism 8.0 software.

### 2.8. Ethics Statement

The study was approved by the International Centre for Genetic Engineering and Biotechnology’s institutional ethics committee (ICGEB/IEC/2019/09) dated 6 August 2019.

## 3. Results

### 3.1. Comparison of Dengue Virus Specific Neutralization Titers Using the Standard Focus-Based and Flow Cytometry Based Neutralization Tests

**Figure 1:**
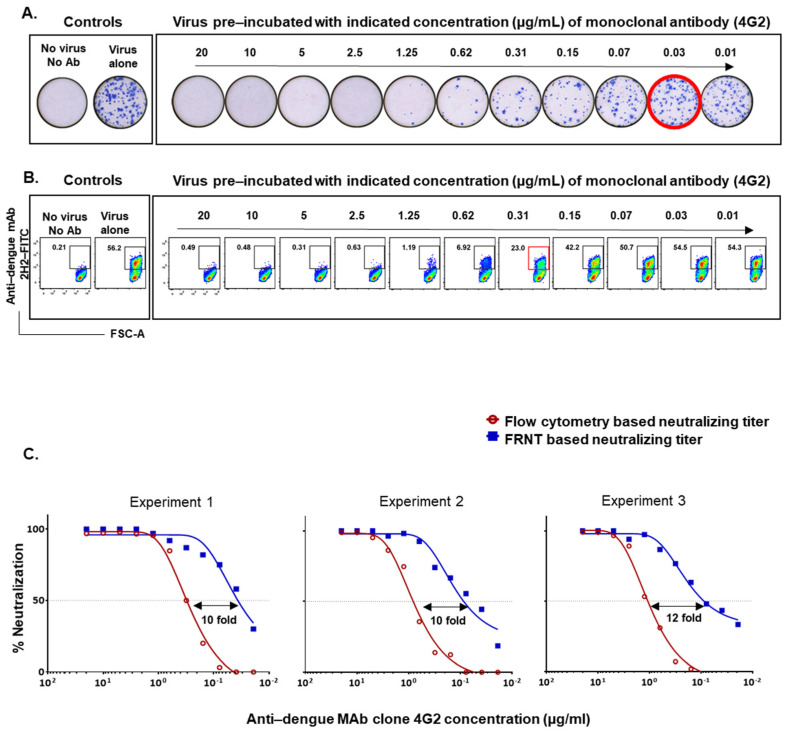
Comparison of neutralization titers obtained by FRNT and flow cytometry based assays using 4G2 monoclonal antibody. (**A**) Raw data on DENV2 neutralization by 4G2 monoclonal antibody in Vero cells using the focus reduction neutralization test (FRNT). Red circles indicate the 50% neutralizing titer (FRNT_50_). (**B**) Raw data on DENV2 neutralization by 4G2 monoclonal antibody in Vero cells using the flow-based neutralization assay. Red box indicates the approximate dilution at which 50% neutralization occurred. (**C**) The horizontal dotted line plotted over the nonlinear dose–response regression analysis indicates the concentration of 4G2 monoclonal antibody needed to cause 50% reduction of neutralization using the FRNT (blue line) and the flow cytometry-based assay (red line) in three independent experiments using different batches of the 4G2 MAb.

The standard protocols typically use around 15,000 Vero cells infected at an MOI of 0.01 for the FRNT assay and around 50,000 Vero cells infected at an MOI of 1 for the flow-based neutralization assay [7,12]. Using these conventional protocols, we first asked how well the neutralization titers obtained by the two assays compared. In the initial experiments, we used anti-flavivirus monoclonal antibody clone 4G2, which is well-established to neutralize all four dengue serotypes [19,20]. We performed a twofold dilution series of the 4G2 antibody starting at 20 µg/mL and incubated with 150 PFU DENV2 to reach an MOI of 0.01 for FRNT, and incubated with 50,000 PFU DENV2 to reach an MOI of 1 for the flow cytometry based assay. Examples of raw data from one of the experiments using graded concentrations of 4G2 monoclonal antibody by FRNT and the flow-cytometry based methods are shown in Figure 1A,B, respectively. A nonlinear dose–response regression analysis of the 4G2 antibody concentration that resulted in a 50% reduction in the neutralization showed that the flow-based assay using the assay conditions described above underestimated the neutralization titers by about 10-fold as compared to FRNT-based titer (Figure 1C). A similar trend was seen in three independent experiments using different batches of the 4G2 MAb. This finding is consistent with a previous study using MAb P3D05 [17].

**Figure 2:**
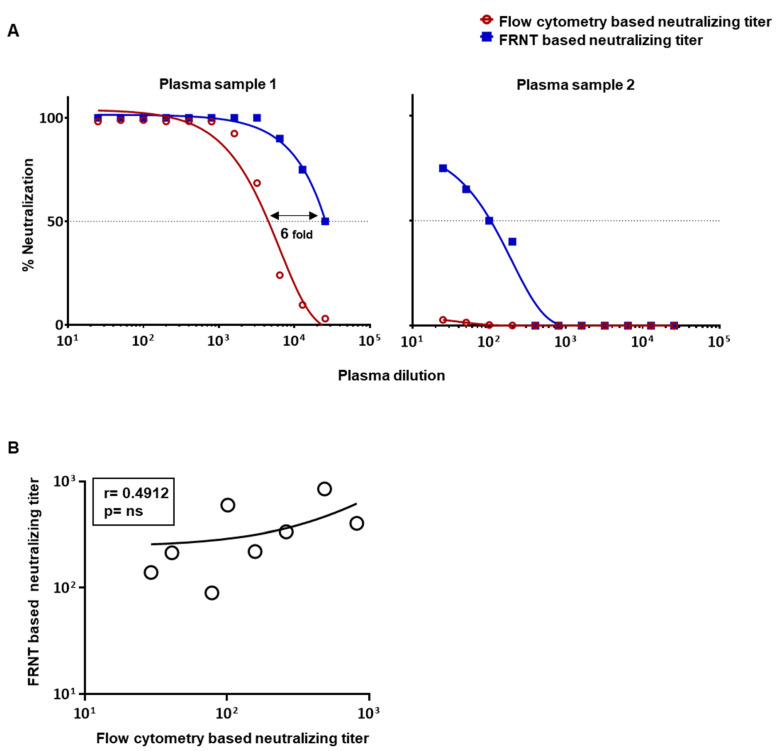
Comparison of neutralization titers obtained by FRNT and flow cytometry based assays in human plasma samples using the standard protocol. (**A**) The horizontal dotted line plotted over the nonlinear dose–response regression analysis indicates the dilution of plasma needed to cause 50% reduction in neutralization of DENV2 using FRNT (blue line) and flow cytometry based assays (red line). Neutralizing assay of plasma samples with very high (plasma sample 1) and very low (plasma sample 2) neutralization titers are shown. (**B**) Correlation analysis of the calculated 50% neutralization titer in an additional 8 individual plasma samples’ intermediate titers using the FRNT- and flow cytometry based assays. Sample 1 and sample 2 shown in panel A are not included in the correlation analysis in panel B due to the outlier nature of these samples and inability to calculate neutralization titer for sample 2 by flow cytometry based assay [23].

We next asked whether the flow cytometry based assay, using the assay conditions described above, would also underestimate neutralization titers in human plasma samples. For this, we initially chose two previously well-characterized plasma samples, one of which had a very high neutralization titer of around 10^4^ and another having a very low neutralization titer of around 10^2^ for dengue virus 2 [13]. Figure 2A shows the comparison of percent neutralization at graded dilutions of the plasma using the two types of assay. Consistent with our findings using the monoclonal antibody, we again found that the flow cytometry based assay vastly underestimated neutralization titers as compared to the FRNT-based titer when we used a highly neutralizing plasma sample (Figure 2A(left)), and even failed to score any neutralization activity when we used plasma with low levels of neutralizing antibodies (Figure 2A(right)). Comparison of additional plasma samples with intermediate neutralization titers further confirmed a poor correlation between these two assays (Figure 2B). These results prompted us to make efforts to further optimize the flow-based assay to improve its sensitivity and ensure that the titers obtained by this method are in better concordance with the titers obtained by the FRNT-based assay.

### 3.2. Optimization of the Flow-Based Assay Such That the Titers Obtained Are in Concordance with the Traditional Foci-Based Neutralizing Assay Titers

The 96-well plate foci-based neutralizing assay typically relies on incubating the test sample with a very small number of viral particles, usually around 100–200 PFU, to allow an MOI of around 0.01 to infect the 15,000 Vero cells seeded per well. This results in visually countable foci. By contrast, it should be noted that the flow cytometry based neutralizing assay typically relies on incubating the test sample with a very high number of viral particles, usually around 50,000 PFU, to allow an MOI of 1 to infect the 50,000 Vero cells seeded per well. Thus, we rationalized that the flow cytometry based assay might be underestimating the titer due to the higher number of viral particles used in the assay. Since the input cell inoculum or viral particle number used for the FRNT was optimized in such a way that the foci would be countable within the well, we wondered whether we could focus on optimizing the flow cytometry based neutralizing assay further such that the neutralization titers obtained by this flow cytometry based assay were in concordance with the traditional foci-based neutralizing assay.

**Figure 3:**
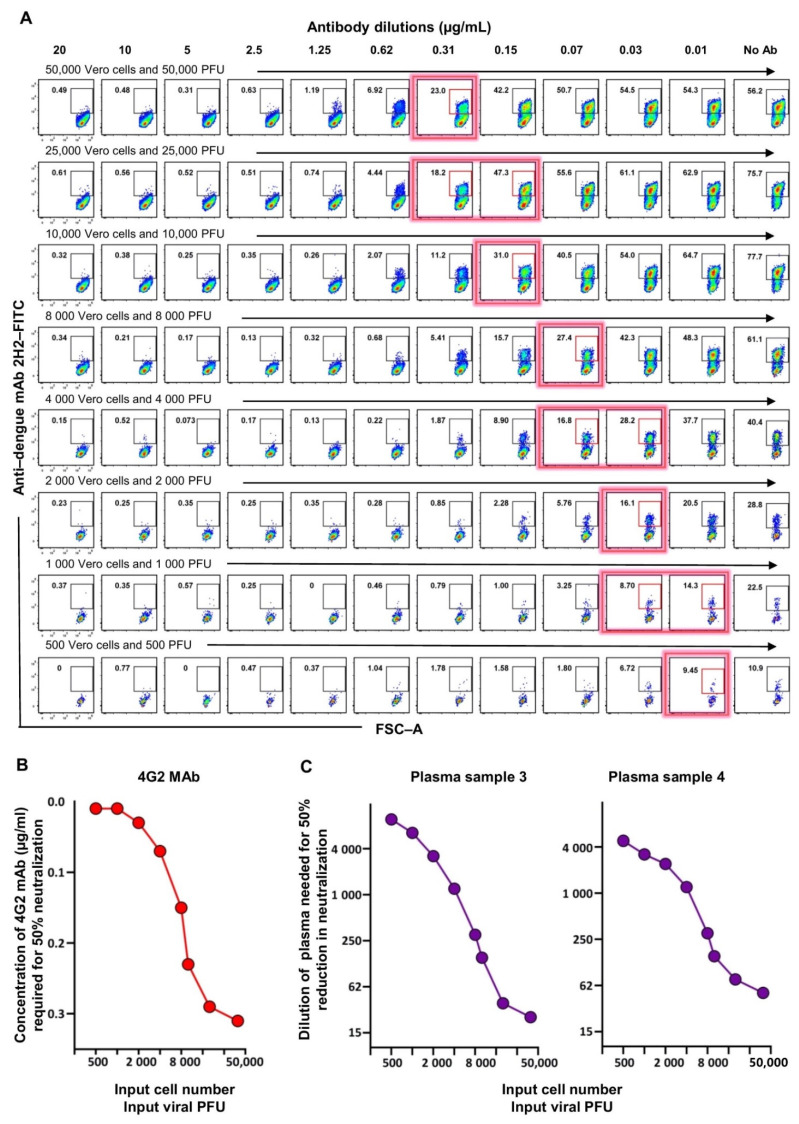
Optimization of the flow cytometry based neutralizing assay such that the neutralization titers obtained by this assay are in concordance with the traditional foci-based neutralizing assay. (**A**) Raw data shown are for flow cytometry staining plots for Vero cells that were seeded at the indicated numbers per well and then infected with the indicated PFU of DENV2 that was preincubated with titrating concentrations of 4G2 monoclonal antibody. Cells were stained 24 h post-infection using anti-dengue 2H2 monoclonal antibody. Red box and red shadow on a flow cytometry plot indicate the approximate position where 50% neutralization of virus was achieved. (**B**) Concentration of 4G2 monoclonal antibody needed to cause 50% neutralization in the flow cytometry based assay using graded number of Vero cells at an MOI of 1. (**C**) Dilution of plasma needed to cause 50% neutralization in the flow cytometry based assay using graded number of Vero cells at an MOI of 1. Two individual plasma samples are shown.

We rationalized that we could be able to achieve this by decreasing the viral inoculum for the flow-based assay to a PFU level closer to what is used in the FRNT since neutralization potency may be affected by the abundance of the viral particles. However, we recognized that decreasing the viral PFU alone decreases the MOI as well, thereby leading to a substantial drop in the percent of cells that are infected, thus complicating the assay. Therefore, we chose to decrease both the input Vero cell number as well as the PFU of the virus in such a way that the MOI of 1 remained constant. We then evaluated a series of graded numbers of Vero cells along with the corresponding titer of the dengue virus (i.e., 50,000 Vero cells with 50,000 PFU virus, 25,000 Vero cells with 25,000 PFU virus, and so on). Examples of raw data using these different combinations with 4G2 MAb as test samples are shown in Figure 3A, and the concentrations of the 4G2 Ab needed for 50% inhibition of DENV2 at each Vero cell input or viral inoculum level are shown in Figure 3B. Consistent with the rationale described above, we observed that, as the input cell number and the input viral inoculum was proportionately lowered, keeping virus MOI constant at 1, the concentration of the 4G2 antibody required for 50% neutralization became lower (Figure 3B). In other words, the assay sensitivity became better by simultaneously lowering the input Vero cell number and the input viral inoculum. Moreover, we found that an input Vero cell number of 2000 of cells per well together with a viral titer of 2000 PFU, to give an MOI of 1, brought the concentration of the 4G2 antibody needed for 50% neutralization by flow cytometry based assay very close to the concentration of the 4G2 antibody needed for 50% neutralization by FRNT (0.03 ug/mL). We then replicated this analysis using two different plasma samples and observed a similar trend (Figure 3C).

### 3.3. Evaluation of Correlation between the Neutralizing Titers by the Optimized Flow-Based Neutralizing Assay versus the Traditional Foci Reduction Neutralizing Assay

**Figure 4:**
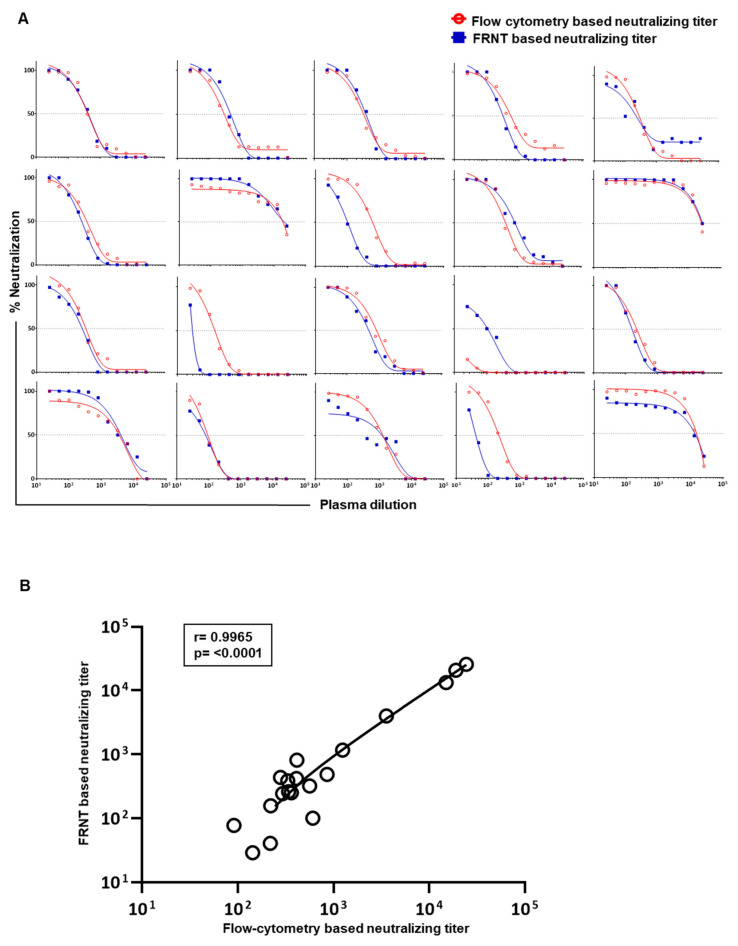
Correlation analysis between the neutralization titers obtained by the newly improved flow cytometry based assay and the standard FRNT. (**A**) Nonlinear dose–response regression analysis of neutralization at different dilutions of plasma from 20 individuals is shown for FRNT (blue line) and flow cytometry based assays (red line). Flow cytometry based neutralization assays were performed using 2000 Vero cells/well at an MOI of 1. (**B**) Graph shows correlation analysis of the calculated 50% neutralization for DENV2 in plasma samples shown in A. The sample indicated in the fourth row of third column of panel A is excluded in this correlation analysis due to inability to calculate the neutralization titer of this particular sample by the flow cytometry based method.

**Figure 5:**
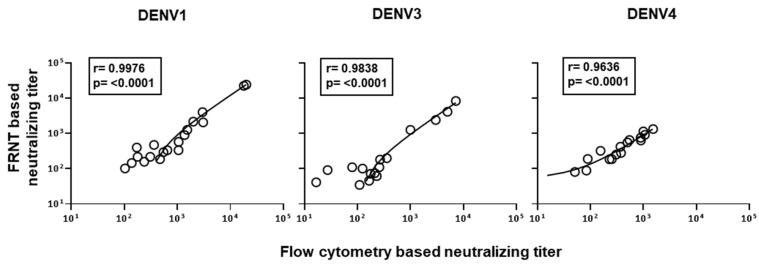
Correlation analysis between the neutralization titers obtained by the newly improved flow cytometry based assays and the standard FRNT for DENV1, DENV3, and DENV4 serotypes. Flow cytometry based neutralization assays were performed using 2000 Vero cells/well at an MOI of 1. Correlation analysis of DENV-1, 3, and 4 serotypes are shown in left, middle, and right graph, respectively.

Considering the above findings, we settled on 2000 Vero cells and 2000 PFU virus for the flow cytometry based assay to further scrutinize the correlation of neutralization titers obtained by this improved flow cytometry based neutralizing assay versus the traditional gold-standard foci reduction neutralizing assay. Raw data on neutralization curves generated against DENV2 in 20 individual plasma samples using both assays are shown in Figure 4A. Using this improved protocol for the flow cytometry based assay, we observed a strong concordance (*r* = 0.9965, *p* < 0.0001) with the neutralization titers obtained by both the modified flow cytometry based and traditional foci-based assays (Figure 4B). Similar results were obtained for neutralization titers against DENV1, DENV3, and DENV4 serotypes (Figure 5).

## 4. Discussion

In summary, our study provides an optimized methodology for performing a high-throughput flow cytometry based assay to measure neutralizing antibody responses against each of the four dengue virus serotypes in such a way that the neutralization titers obtained by this assay closely match with the titers obtained by the traditional foci-based neutralizing assay. Given that dengue is a global public health threat and that several research groups are making efforts to understand its pathophysiology and engage in vaccine development evaluation in India and worldwide [5,24,25], our findings have timely significance for facilitating these efforts.

Although the traditional PRNT/FRNT assays are generally considered gold-standard assays for detecting neutralizing titers against dengue virus, they also have several disadvantages—they are labor intensive, time consuming, and subjective to interpretation of the plaques/foci formed. The high-throughput flow cytometry based neutralization assay described here, while ensuring that the titers obtained by this method are in concordance with the titers by the FRNT method, makes it possible to overcome most of these drawbacks and thus has the potential to become the method of choice to screen the large number of samples generated during vaccine evaluation trials. It should be noted that the neutralization titers reported in the literature can change from one laboratory to another depending on the type and number of host cells, the concentration of virus inoculum, and the duration and type of the assay employed. A literature search of anti-flavivirus monoclonal antibody 4G2 that is well-established to neutralize all four dengue serotypes is reported to have a 50% neutralization titer that ranges from 0.1 µg/mL to 5 µg/mL [19,20,26,27]. To this end, it is extremely beneficial to have a robustly standardized high-throughput flow cytometry based neutralization assay that gives titers which are in concordance with the gold-standard plaque/foci-based assays that have been typically used to evaluate vaccines. The modified protocol described here for the flow cytometry based assay is thus significant.

Interestingly, our study also highlights that the neutralization titers calculated in the in vitro assays can vary substantially depending on the available target cell number and viral inoculum. This is important to note since there is no uniformity in the number of Vero cells used for flow cytometry based neutralization assays described for dengue in the literature so far [7,16]. It is also important to note that neutralization assays are generally performed using Fc gamma receptor (FcγR)-deficient cell lines, such as Vero cells, as used in our study and others [7,12,13,26]. These FcγR-deficient cell lines are suitable for the detection of neutralization rather than antibody-dependent enhancement (ADE). On the other hand, FcγR- positive cells, which are typically used for ADE assays, can serve as potential targets for both direct infection and ADE. Since the ADE assays are generally based on flow cytometry and use high virus MOI (typically MOI 1) [22,28,29], it would be interesting to address how the ADE titers behave if the cell numbers and viral inoculum used for ADE assays are adjusted to match the cell numbers and viral inoculum used in the improved flow cytometry based neutralization assay described here. Further studies are warranted to address these issues.

Our study is limited to the analysis of the correlation of neutralization titers obtained by flow cytometry versus foci-based methods using well-characterized monoclonal antibodies or plasma samples derived from previously exposed healthy subjects in a dengue-endemic setting. How well these assays correlate if the test samples are derived from acute febrile patients or vaccines remains to be determined. Additionally, further studies are warranted to stringently evaluate the inter-laboratory variability of the improved high-throughput flow cytometry based neutralizing assay methodology described in our study to support dengue epidemiology and vaccine evaluation efforts in various parts of the world in a standardized manner.

In conclusion, as dengue vaccines continue to be evaluated in India and other parts of the word, a high-throughput assay that allows for the screening of a large number of samples is critically needed. Our revised protocol of a 96-well plate flow cytometry based neutralizing assay fulfills the abovesaid purpose, while simultaneously having strong concordance with the traditional neutralizing assays.

## Data Availability

Not applicable.
